# 2-(4-Bromo­phen­oxy)propanohydrazide

**DOI:** 10.1107/S1600536809003134

**Published:** 2009-01-31

**Authors:** Tashfeen Akhtar, M. Khawar Rauf, Masahiro Ebihara, Shahid Hameed

**Affiliations:** a Department of Chemistry, Quaid-i-Azam University, Islamabad 45320, Pakistan; b Department of Chemistry, Faculty of Engineering, Gifu University, Yanagido, Gifu 501-1193, Japan

## Abstract

The title compound, C_9_H_11_BrN_2_O_2_, is an important inter­mediate for the synthesis of heterocyclic compounds such as azoles, 2,5-disubstituted-1,3,4-oxadiazo­les and 5-substituted 2-mercapto-1,3,4-oxadiazo­les. The bromo­phen­oxy group subtends a dihedral angle of 82.81 (7)° with the plane passing through the propanohydrazide moiety. The crystal structure is stabilized by inter­molecular N—H⋯O hydrogen bonds that form columns extending along the *b* axis.

## Related literature

For carboxy­hydrazide derivatives with biological activities, see: Belkadi & Othman (2006 ▶); Goswami *et al.* (1984 ▶); Akhtar *et al.* (2008 ▶); Akhtar, Hameed *et al.* (2007 ▶); Ahmad *et al.* (1996 ▶); Akhtar *et al.* (2006 ▶); For related structures, see: Akhtar, Khawar Rauf *et al.* (2007 ▶); Zheng (2008 ▶).
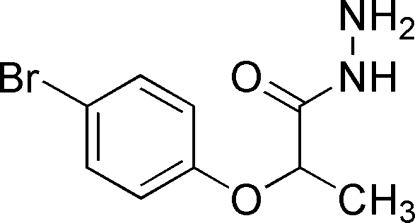


## Experimental

### 

#### Crystal data

C_9_H_11_BrN_2_O_2_
*M* *_r_* = 259.11
Monoclinic, 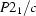

*a* = 10.2598 (14) Å
*b* = 4.8009 (7) Å
*c* = 23.322 (3) Å
β = 112.712 (6)°
*V* = 1059.7 (3) Å^3^
*Z* = 4
Mo *K*α radiation
μ = 3.86 mm^−1^
*T* = 113 (2) K
0.50 × 0.30 × 0.20 mm

#### Data collection

Rigaku/MSC Mercury CCD diffractometer
Absorption correction: integration (*NUMABS*; Higashi, 1999) *T*_min_ = 0.531, *T*_max_ = 0.759
8296 measured reflections
2418 independent reflections
2201 reflections with *I* > 2σ(*I*)
*R*_int_ = 0.039

#### Refinement

*R*[*F* ^2^ > 2σ(*F* ^2^)] = 0.048
*wR*(*F* ^2^) = 0.076
*S* = 1.20
2418 reflections
137 parameters
H atoms treated by a mixture of independent and constrained refinement
Δρ_max_ = 0.59 e Å^−3^
Δρ_min_ = −0.75 e Å^−3^

### 

Data collection: *CrystalClear* (Molecular Structure Corporation & Rigaku, 2001 ▶); cell refinement: *CrystalClear*; data reduction: *TEXSAN* (Molecular Structure Corporation & Rigaku, 2004 ▶); program(s) used to solve structure: *SIR97* (Altomare *et al.*, 1999 ▶); program(s) used to refine structure: *SHELXL97* (Sheldrick, 2008 ▶); molecular graphics: *ORTEPII* (Johnson, 1976 ▶); software used to prepare material for publication: *SHELXL97* and *TEXSAN*.

## Supplementary Material

Crystal structure: contains datablocks I, global. DOI: 10.1107/S1600536809003134/si2151sup1.cif

Structure factors: contains datablocks I. DOI: 10.1107/S1600536809003134/si2151Isup2.hkl

Additional supplementary materials:  crystallographic information; 3D view; checkCIF report

## Figures and Tables

**Table 1:** Hydrogen-bond geometry (Å, °)

*D*—H⋯*A*	*D*—H	H⋯*A*	*D*⋯*A*	*D*—H⋯*A*
N1—H1⋯O1^i^	0.85 (3)	1.97 (3)	2.812 (3)	170 (3)
N2—H2*A*⋯O1^ii^	0.83 (3)	2.33 (3)	3.127 (3)	161 (3)

Symmetry codes: (i) 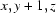; (ii) 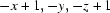.

**Fig. 1.**
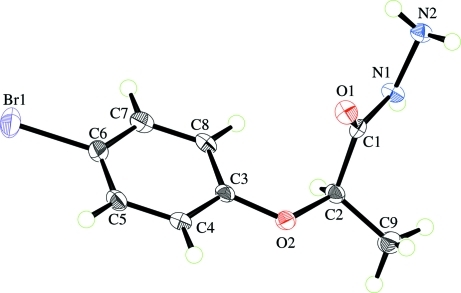
The molecular structure of (I) showing the atom labelling and displacement ellipsoids drawn at the 50% probability level.

**Fig. 2.**
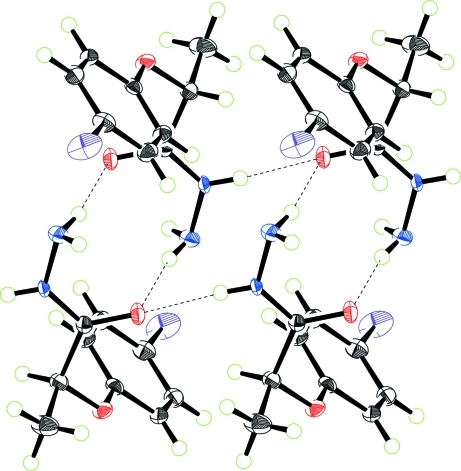
View of the N—H···O hydrogen bonded molecules. The unit cell has been omitted for clarity.

## supplementary crystallographic information

### Comment

Carboxylic acid hydrazides are important biological agents and intermediates in the synthesis of biologically active heterocycles with two nitrogen atoms at adjacent positions (Belkadi & Othman, 2006). The hydrazides when treated with isocyanates or isothiocyanates afford semicarbazides and thiosemicarbazides, respectively (Goswami *et al.*, 1984). These are important intermediates in the synthesis of azoles under acidic or basic conditions (Akhtar *et al.*, 2007*a*; Ahmad *et al.*, 1996). In continuation of our previous studies (Akhtar *et al.*, 2006; Akhtar *et al.*, 2007*b*), the title compound, 2-(4-bromophenoxy)propane hydrazide,was synthesized as an intermediate in the synthesis of certain azole derivatives (Akhtar *et al.*, 2008). The C—N bond length of 1.330 (3)Å is similar to C—N 1.321 (3) Å, indicating the single bond character.The N1—N2 bond length of 1.415 (3) Å in the title compound is longer than the N—N distance [1.366 (3)Å] in the crystal structure of *N*-propionyl-*N'*-(3-hydroxy-2-naphthoyl)hydrazide (Zheng, 2008). The *Bromo* group is coplanar with the phenyl plane C3/C4/C5/C6/C7/C8 with deviation from the plane of 0.030 (4) Å. The molecular packing diagram (Fig. 2) shows the presence of two intermolecular N—H···O hydrogen bonds, (Table 1), one of which is generated *via* translation along [0 1 0], the other *via* inversion symmetry.

### Experimental

Methyl 2-(4-bromophenoxy)propionate (5.0 g, 0.0193 mol) was dissolved in methanol (20 ml) and hydrazine hydrate (80%, 3.50 mL, 0.0679 mol) added slowly with stirring. The reaction mixture was set to reflux. After completion of the reaction (TLC, 6 hrs), the reaction mixture was concentrated and poured to water. The precipitated solid was filtered and recrystallized from ethanol/ water. The spectroscopic and physical characterization data will be reported separately.

### Refinement

The H atoms on the N atoms were refined isotropically. Other H atoms were placed in idealized positions and treated as riding atoms with C—H distance in the range 0.95–1.000 Å and *U*_iso_(H) = 1.2*U*_eq_(C) or 1.5*U*_eq_(C).

### Crystal data

**Table:**

C_9_H_11_BrN_2_O_2_	*F*(000) = 520
*M**_r_* = 259.11	*D*_x_ = 1.624 Mg m^−^^3^
Monoclinic, *P*2_1_/*c*	Mo *K*α radiation, λ = 0.7107 Å
Hall symbol: -P 2ybc	Cell parameters from 2804 reflections
*a* = 10.2598 (14) Å	θ = 3.4–27.5°
*b* = 4.8009 (7) Å	µ = 3.86 mm^−^^1^
*c* = 23.322 (3) Å	*T* = 113 K
β = 112.712 (6)°	Block, colorless
*V* = 1059.7 (3) Å^3^	0.50 × 0.30 × 0.20 mm
*Z* = 4	

### Data collection

**Table:**

Rigaku/MSC Mercury CCD diffractometer	2201 reflections with *I* > 2σ(*I*)
Detector resolution: 14.62 pixels mm^-1^	*R*_int_ = 0.039
ω scans	θ_max_ = 27.5°, θ_min_ = 3.4°
Absorption correction: integration (*NUMABS*; Higashi, 1999)	*h* = −13→11
*T*_min_ = 0.531, *T*_max_ = 0.759	*k* = −6→4
8296 measured reflections	*l* = −26→30
2418 independent reflections	

### Refinement

**Table:**

Refinement on *F*^2^	Primary atom site location: structure-invariant direct methods
Least-squares matrix: full	Secondary atom site location: difference Fourier map
*R*[*F*^2^ > 2σ(*F*^2^)] = 0.048	Hydrogen site location: inferred from neighbouring sites
*wR*(*F*^2^) = 0.076	H atoms treated by a mixture of independent and constrained refinement
*S* = 1.20	*w* = 1/[σ^2^(*F*_o_^2^) + (0.0037*P*)^2^ + 1.6325*P*] where *P* = (*F*_o_^2^ + 2*F*_c_^2^)/3
2418 reflections	(Δ/σ)_max_ = 0.001
137 parameters	Δρ_max_ = 0.59 e Å^−^^3^
0 restraints	Δρ_min_ = −0.75 e Å^−^^3^

### Special details

**Table:**

Geometry. All esds (except the esd in the dihedral angle between two l.s. planes) are estimated using the full covariance matrix. The cell esds are taken into account individually in the estimation of esds in distances, angles and torsion angles; correlations between esds in cell parameters are only used when they are defined by crystal symmetry. An approximate (isotropic) treatment of cell esds is used for estimating esds involving l.s. planes.
Refinement. Refinement of *F*^2^ against ALL reflections. The weighted *R*-factor *wR* and goodness of fit *S* are based on *F*^2^, conventional *R*-factors *R* are based on *F*, with *F* set to zero for negative *F*^2^. The threshold expression of *F*^2^ > σ(*F*^2^) is used only for calculating *R*-factors(gt) *etc*. and is not relevant to the choice of reflections for refinement. *R*-factors based on *F*^2^ are statistically about twice as large as those based on *F*, and *R*- factors based on ALL data will be even larger.

### Fractional atomic coordinates and isotropic or equivalent isotropic displacement parameters (Å^2^)

**Table:**

	*x*	*y*	*z*	*U*_iso_*/*U*_eq_	
C1	0.4780 (3)	0.1909 (5)	0.40801 (12)	0.0143 (5)	
O1	0.4868 (2)	−0.0616 (4)	0.41874 (9)	0.0203 (4)	
N1	0.5635 (2)	0.3771 (4)	0.44658 (11)	0.0162 (5)	
H1	0.551 (3)	0.551 (6)	0.4399 (14)	0.019*	
N2	0.6750 (3)	0.3047 (5)	0.50307 (11)	0.0200 (5)	
H2A	0.643 (3)	0.205 (7)	0.5240 (15)	0.024*	
H2B	0.740 (3)	0.209 (6)	0.4928 (14)	0.024*	
C2	0.3698 (3)	0.3120 (6)	0.34780 (13)	0.0188 (6)	
H2	0.3260	0.4839	0.3568	0.023*	
O2	0.2635 (2)	0.1095 (4)	0.31761 (9)	0.0199 (4)	
C3	0.1643 (3)	0.0534 (5)	0.34207 (13)	0.0164 (5)	
C4	0.0672 (3)	−0.1518 (6)	0.31070 (12)	0.0180 (5)	
H4	0.0761	−0.2460	0.2766	0.022*	
C5	−0.0425 (3)	−0.2199 (6)	0.32900 (13)	0.0205 (6)	
H5	−0.1092	−0.3595	0.3076	0.025*	
C6	−0.0528 (3)	−0.0808 (6)	0.37882 (14)	0.0222 (6)	
C7	0.0439 (3)	0.1215 (6)	0.41093 (14)	0.0218 (6)	
H7	0.0353	0.2140	0.4453	0.026*	
C8	0.1534 (3)	0.1881 (6)	0.39257 (13)	0.0190 (6)	
H8	0.2208	0.3256	0.4145	0.023*	
Br1	−0.20430 (4)	−0.16915 (9)	0.403569 (18)	0.04154 (13)	
C9	0.4395 (4)	0.3772 (7)	0.30247 (15)	0.0332 (8)	
H9A	0.4827	0.2076	0.2943	0.050*	
H9B	0.5125	0.5194	0.3204	0.050*	
H9C	0.3682	0.4466	0.2634	0.050*	

### Atomic displacement parameters (Å^2^)

**Table:**

	*U*^11^	*U*^22^	*U*^33^	*U*^12^	*U*^13^	*U*^23^
C1	0.0158 (13)	0.0134 (12)	0.0163 (13)	−0.0004 (10)	0.0090 (11)	0.0010 (10)
O1	0.0227 (11)	0.0116 (9)	0.0254 (11)	−0.0004 (8)	0.0079 (9)	0.0015 (8)
N1	0.0194 (12)	0.0079 (10)	0.0167 (12)	0.0010 (9)	0.0020 (10)	0.0014 (9)
N2	0.0196 (12)	0.0209 (12)	0.0168 (12)	0.0003 (10)	0.0041 (10)	0.0026 (10)
C2	0.0176 (13)	0.0184 (13)	0.0176 (14)	−0.0041 (11)	0.0035 (11)	0.0023 (11)
O2	0.0209 (10)	0.0220 (10)	0.0152 (10)	−0.0090 (8)	0.0053 (8)	−0.0031 (8)
C3	0.0145 (13)	0.0166 (13)	0.0146 (13)	0.0008 (10)	0.0017 (11)	0.0041 (10)
C4	0.0186 (13)	0.0177 (13)	0.0140 (13)	0.0000 (11)	0.0023 (11)	−0.0006 (11)
C5	0.0178 (14)	0.0194 (14)	0.0194 (15)	−0.0039 (11)	0.0018 (11)	0.0008 (11)
C6	0.0165 (14)	0.0272 (15)	0.0218 (15)	0.0005 (11)	0.0062 (12)	0.0047 (12)
C7	0.0212 (15)	0.0216 (15)	0.0200 (15)	0.0031 (11)	0.0049 (12)	−0.0021 (11)
C8	0.0159 (13)	0.0182 (13)	0.0176 (14)	−0.0010 (11)	0.0006 (11)	−0.0019 (11)
Br1	0.02723 (18)	0.0649 (3)	0.0383 (2)	−0.01613 (17)	0.01901 (15)	−0.01174 (19)
C9	0.0321 (18)	0.043 (2)	0.0216 (16)	−0.0163 (15)	0.0075 (14)	0.0044 (14)

### Geometric parameters (Å, °)

**Table:**

C1—O1	1.234 (3)	C4—C5	1.389 (4)
C1—N1	1.330 (3)	C4—H4	0.9500
C1—C2	1.529 (4)	C5—C6	1.379 (4)
N1—N2	1.415 (3)	C5—H5	0.9500
N1—H1	0.85 (3)	C6—C7	1.384 (4)
N2—H2A	0.83 (3)	C6—Br1	1.903 (3)
N2—H2B	0.92 (3)	C7—C8	1.385 (4)
C2—O2	1.427 (3)	C7—H7	0.9500
C2—C9	1.520 (4)	C8—H8	0.9500
C2—H2	1.0000	C9—H9A	0.9800
O2—C3	1.372 (3)	C9—H9B	0.9800
C3—C8	1.385 (4)	C9—H9C	0.9800
C3—C4	1.392 (4)		
			
O1—C1—N1	123.1 (2)	C5—C4—H4	119.8
O1—C1—C2	122.0 (2)	C3—C4—H4	119.8
N1—C1—C2	114.9 (2)	C6—C5—C4	118.7 (3)
C1—N1—N2	123.4 (2)	C6—C5—H5	120.7
C1—N1—H1	121 (2)	C4—C5—H5	120.7
N2—N1—H1	115 (2)	C5—C6—C7	121.6 (3)
N1—N2—H2A	109 (2)	C5—C6—Br1	119.0 (2)
N1—N2—H2B	107 (2)	C7—C6—Br1	119.4 (2)
H2A—N2—H2B	111 (3)	C6—C7—C8	119.5 (3)
O2—C2—C9	105.8 (2)	C6—C7—H7	120.3
O2—C2—C1	109.9 (2)	C8—C7—H7	120.3
C9—C2—C1	110.3 (2)	C3—C8—C7	119.8 (3)
O2—C2—H2	110.3	C3—C8—H8	120.1
C9—C2—H2	110.3	C7—C8—H8	120.1
C1—C2—H2	110.3	C2—C9—H9A	109.5
C3—O2—C2	118.5 (2)	C2—C9—H9B	109.5
O2—C3—C8	125.4 (2)	H9A—C9—H9B	109.5
O2—C3—C4	114.6 (2)	C2—C9—H9C	109.5
C8—C3—C4	120.1 (3)	H9A—C9—H9C	109.5
C5—C4—C3	120.4 (3)	H9B—C9—H9C	109.5
			
O1—C1—N1—N2	1.5 (4)	O2—C3—C4—C5	−177.1 (2)
C2—C1—N1—N2	−176.7 (2)	C8—C3—C4—C5	1.1 (4)
O1—C1—C2—O2	15.9 (4)	C3—C4—C5—C6	−0.2 (4)
N1—C1—C2—O2	−165.9 (2)	C4—C5—C6—C7	−0.6 (4)
O1—C1—C2—C9	−100.3 (3)	C4—C5—C6—Br1	179.2 (2)
N1—C1—C2—C9	77.9 (3)	C5—C6—C7—C8	0.4 (4)
C9—C2—O2—C3	−166.8 (2)	Br1—C6—C7—C8	−179.3 (2)
C1—C2—O2—C3	74.1 (3)	O2—C3—C8—C7	176.8 (2)
C2—O2—C3—C8	3.5 (4)	C4—C3—C8—C7	−1.3 (4)
C2—O2—C3—C4	−178.3 (2)	C6—C7—C8—C3	0.5 (4)

### Hydrogen-bond geometry (Å, °)

**Table:**

*D*—H···*A*	*D*—H	H···*A*	*D*···*A*	*D*—H···*A*
N1—H1···O1^i^	0.85 (3)	1.97 (3)	2.812 (3)	170 (3)
N2—H2A···O1^ii^	0.83 (3)	2.33 (3)	3.127 (3)	161 (3)

Symmetry codes: (i) *x*, *y*+1, *z*; (ii) −*x*+1, −*y*, −*z*+1.
